# MiRNA profiling of gastrointestinal stromal tumors by next-generation sequencing

**DOI:** 10.18632/oncotarget.16664

**Published:** 2017-03-29

**Authors:** Ugne Gyvyte, Simonas Juzenas, Violeta Salteniene, Juozas Kupcinskas, Lina Poskiene, Laimutis Kucinskas, Sonata Jarmalaite, Kristina Stuopelyte, Ruta Steponaitiene, Georg Hemmrich-Stanisak, Matthias Hübenthal, Alexander Link, Sabine Franke, Andre Franke, Dalia Pangonyte, Vaiva Lesauskaite, Limas Kupcinskas, Jurgita Skieceviciene

**Affiliations:** ^1^ Institute for Digestive Research, Academy of Medicine, Lithuanian University of Health Sciences, Kaunas, Lithuania; ^2^ Department of Gastroenterology, Academy of Medicine, Lithuanian University of Health Sciences, Kaunas, Lithuania; ^3^ Department of Pathological Anatomy, Academy of Medicine, Lithuanian University of Health Sciences, Kaunas, Lithuania; ^4^ Division of Human Genome Research Centre, Institute of Biosciences, Life Sciences Center, Vilnius University, Vilnius, Lithuania; ^5^ National Cancer Institute, Vilnius, Lithuania; ^6^ Institute of Clinical Molecular Biology, Christian-Albrechts-University Kiel, Kiel, Germany; ^7^ Department of Gastroenterology, Hepatology and Infectious Diseases, Otto-von-Guericke University Hospital Magdeburg, Magdeburg, Germany; ^8^ Institute of Pathology, Otto-von-Guericke University, Magdeburg, Germany; ^9^ Institute of Cardiology, Academy of Medicine, Lithuanian University of Health Sciences, Kaunas, Lithuania

**Keywords:** GIST, microRNA, microRNA profiling, small RNA-seq

## Abstract

Deregulation of miRNAs has been observed virtually in all major types of cancer, whereas the miRNA signature in GIST is not well characterized yet. In this study the first high-throughput miRNA profiling of 15 paired GIST and adjacent normal tissue samples was performed using small RNA-seq approach and differentially expressed miRNAs as well as isomiRNAs were defined. Highly significantly deregulated miRNAs were selected for validation by Taq-Man low-density array in replication group of 40 paired samples. Validated miRNAs were further subjected to enrichment analysis, which revealed significantly enriched KEGG pathways in the main GIST associated pathways. Further, we used an integrated analysis of miRNA-mRNA correlations for *KIT* and *PDGFRA* target genes and found a significant correlation between all of the enriched miRNAs and their target gene *KIT*. Results of the phenotype analysis showed miR-509-3p to be up-regulated in epithelioid and mixed cell types compared to spindle type, whereas miR-215-5p showed negative correlation with risk grade of GIST. These data reveal a detailed miRNA profile of GIST and highlight new candidates that may be important in the development of malignant disease.

## INTRODUCTION

Gastrointestinal stromal tumors (GISTs), the most common mesenchymal tumors of the gastrointestinal tract, result from constitutively activating mutations in two oncogenes from the receptor tyrosine kinase family, *KIT* or platelet-derived growth factor receptor-α (*PDGFRA*) [1, 2]. GISTs arise in the muscular layer of the tissue and can occur in any part of the gastrointestinal tract, with a predominance of 2/3 cases to be located in the stomach [3]. The clinical behavior of GIST is highly variable from benign to malignant with widespread metastases. Although most GISTs are characterized by *KIT* and *PDGFRA* gene mutations, it is strongly speculated that changes in gene expression are responsible for their gradual malignant transformation, however, little is known about the underlying mechanisms regulating these expression signatures [4, 5].

MicroRNAs (miRNAs) are a class of small non-coding RNAs involved in post-transcriptional regulation of gene expression. MiRNAs are characterized by high stability in biological samples making these molecules an attractive target in the biomarker research field [6, 7]. Previous studies have revealed that deregulation of miRNAs occurs virtually in all major types of cancer, and they are often associated with tumorigenesis, tumor progression, metastasis, and drug resistance pathways [8]. Furthermore, miRNAs have been shown to exhibit a diagnostic or prognostic value and even have potential clinical implications for targeted gene therapy in cancer patients [9–11]. A number of array-based and targeted studies have shown the importance of miRNAs in GIST pathophysiology. Specific miRNA expression signatures have been shown to be associated with chromosome 14q loss [12–14], anatomical site [12, 15], *KIT* or *PDGFRA* mutations [16, 17], tumor risk [12, 18, 19], overall survival [19] and response to treatment [20, 21]. However, to date, a small RNA-seq strategy has not been used for the discovery of GIST-relevant miRNAs.

In our study, we performed the first high-throughput sequencing of small RNAs in a sample set of 15 patients with gastric GIST, focusing on this particular phenotype to lessen the potential impact of clinical variability. We observed distinct miRNA expression profiles between tumor and adjacent tissue samples. MiRNA enrichment analysis indicated that GIST associated miRNAs are involved in the regulation of genes from the main GIST associated pathways. Correlation analysis of miRNA-mRNA revealed a significant correlation between *KIT* and and miRNAs targeting this gene. GIST phenotype analysis showed miR-509-3p to be up-regulated in epithelioid and mixed cell types, whereas miR-215-5p showed negative correlation with risk grade of GIST. The reported findings validate the role of miRNAs in mediating changes that accompany the development of GIST.

## RESULTS

### Global overview of miRNA transcriptome

**Figure 1:**
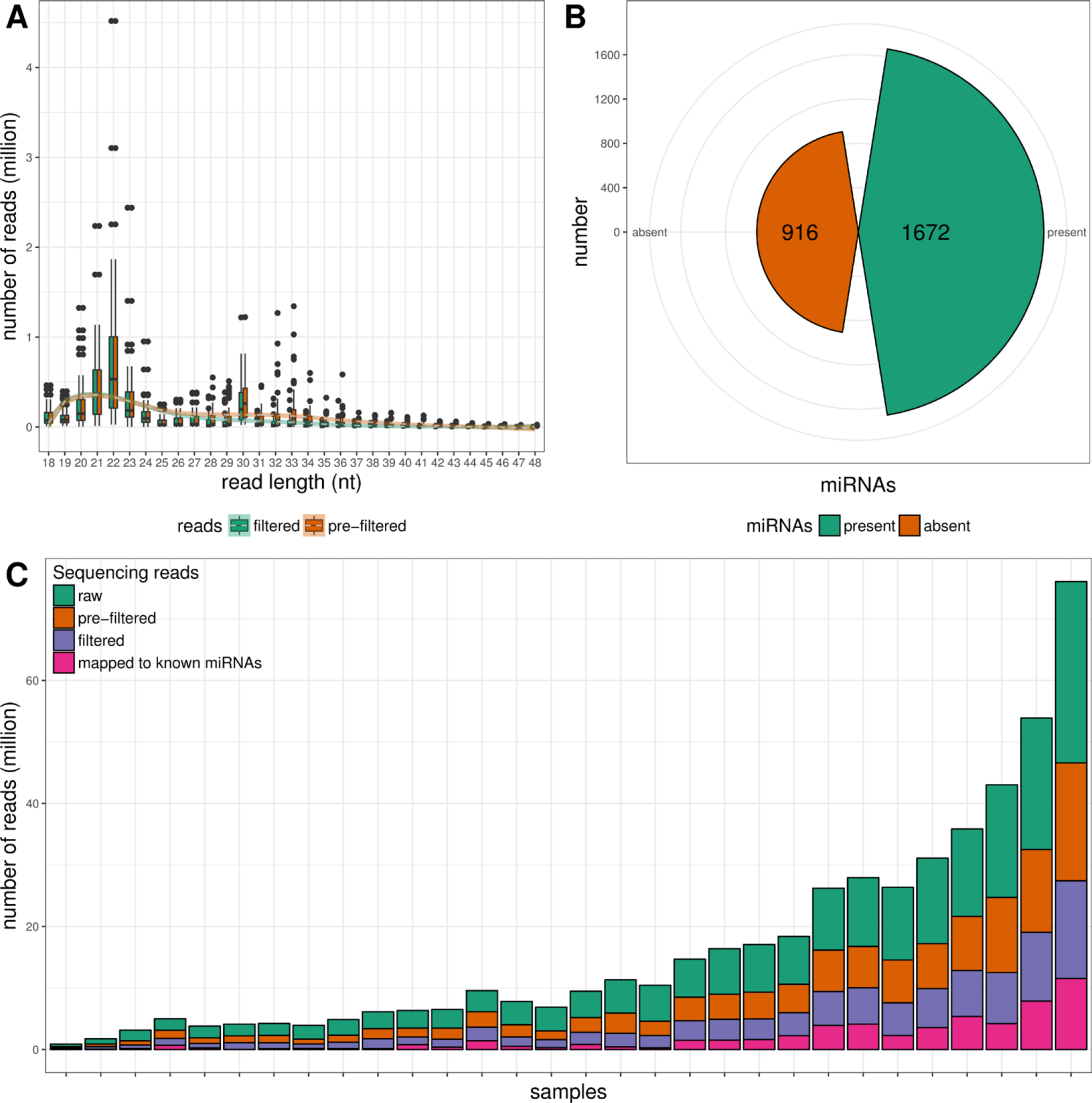
Overview of small RNA NGS data (**A**) Read length distributions of pre-filtered and filtered sequencing reads. (**B**) Number of detected and non-detected known miRNA sequences from miRBase v21. (**C**) Overall composition of processed reads.

Small RNA-seq of 30 formalin-fixed paraffin-embedded (FFPE) tissue samples yielded ~ 271 M raw sequencing reads (from ~417 K to ~29.5 M reads/sample). Pre-filtering and filtering steps retained 50.4% (~137 M) of initial raw reads. The majority of filtered reads were of 20–23 nt length which corresponds to the range of mature miRNA sequences (Figure 1A). Quantification of filtered reads and identification of known miRNAs have yielded ~56 M sequences to be mapped to 1672 known miRNAs from miRBase v21 (Figure 1B). The number of expressed miRNAs ranged from 208 to 1047 per sample. The overall composition of processed reads is shown in Figure 1C.

### Small RNA-seq defines differentially expressed miRNAs in GIST tissues

**Figure 2:**
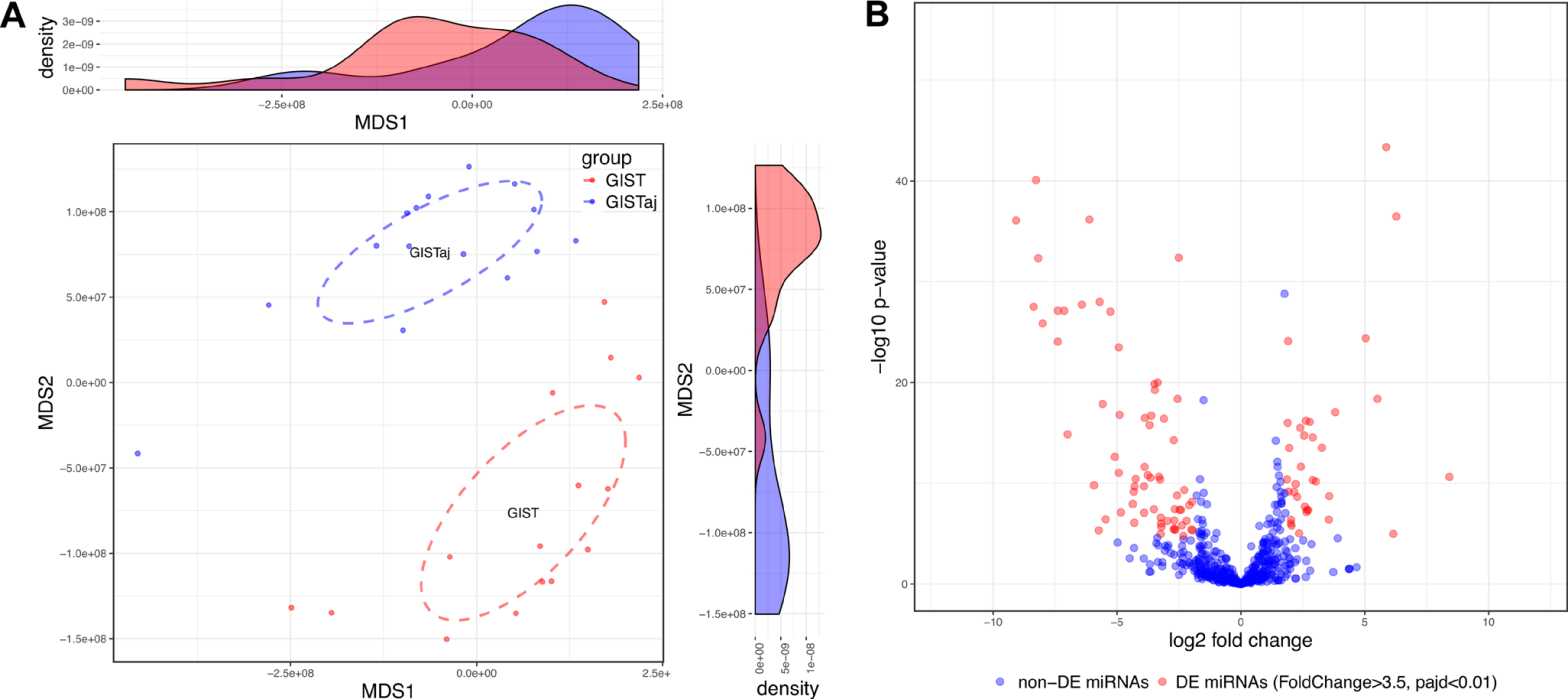
Small RNA-seq defines alterations in miRNAs expression profile of GIST (**A**) MDS plot showing the similarity structure of the miRNA transcriptomes in GIST and their adjacent (GISTaj) tissue samples based on normalized expression values. The dots represent samples colored by group. The centroid of ellipses corresponds to the group mean and the shapes are defined by the covariance within group. The density plots show distributions of the first and second dimensions. (**B**) Volcano plot of aberrantly expressed miRNAs in GIST. The red color represents significantly (Bonferroni adjusted *p*-value < 0.01) differentially expressed miRNAs with fold change < 3.5, while blue color represents non-differentially expressed miRNAs.

**Figure 3:**
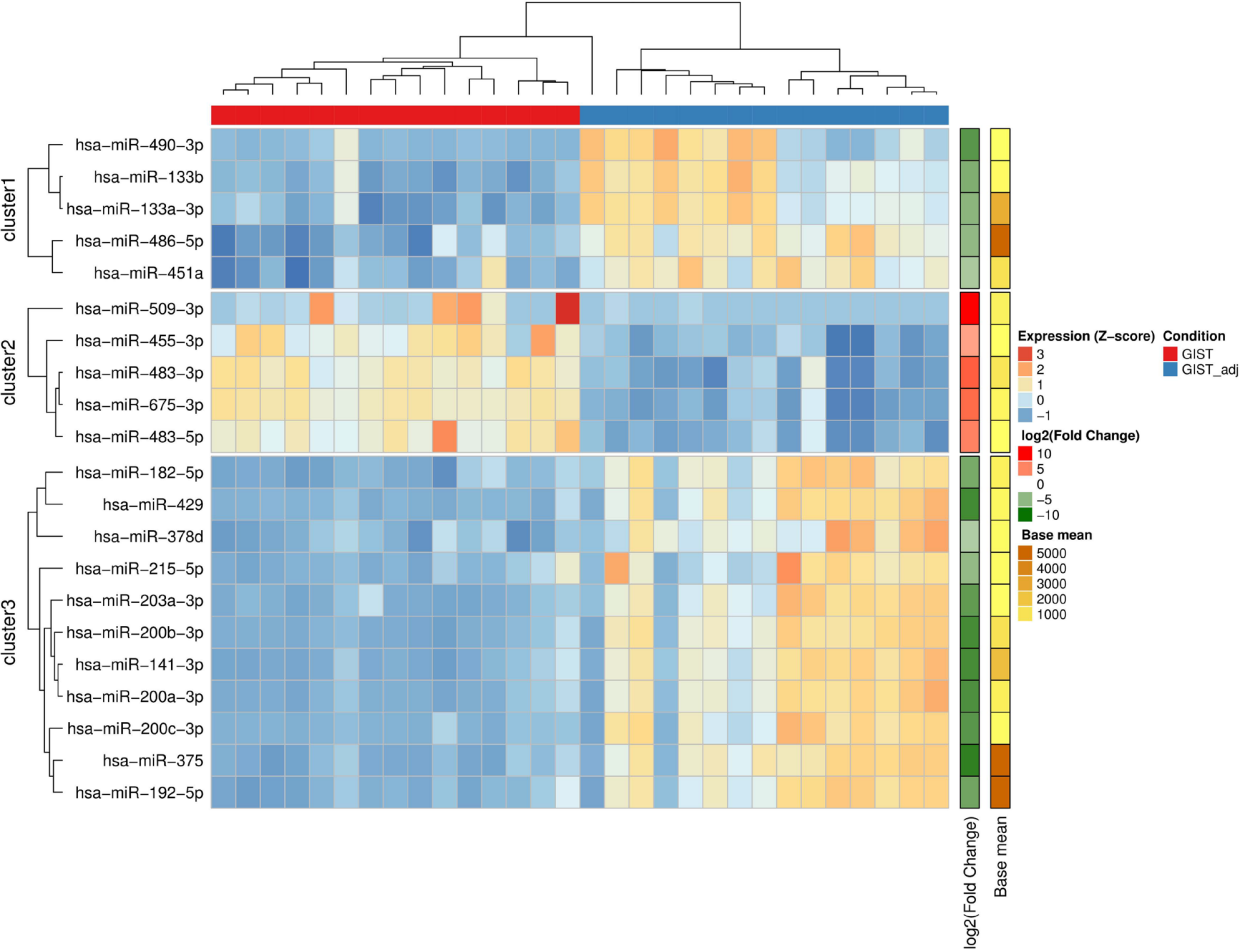
miRNAs discriminate GIST tissues from adjacent non-cancerous tissues Heatmap showing unsupervised hierarchical clustering of normalized miRNA expression levels of GIST and their adjacent (GISTadj) tissue samples based on 21 miRNAs which were selected for validation study. The Z-score represents standardized normalized expression values. The fold changes and abundancies of miRNAs are shown on the right side of the plot.

In order to identify the overall similarity structure of the miRNA transcriptomes, a multidimensional scaling (MDS) analysis using Spearman's correlation distance (1-correlation coefficient) was performed on normalized miRNA count data. The MDS revealed two clearly resolved clusters corresponding to GIST and adjacent non-cancerous tissues (Figure 2A). Paired analysis of normalized miRNA sequencing data identified 100 differentially expressed miRNAs (Bonferroni adjusted *p*-value < 0.01 and fold change > 3.5), 34 of which were up-regulated and 66 were down-regulated (Figure 2B, Supplementary Table 1). For a validation study 21 highly abundant (base mean > 100) and strongly deregulated molecules (Bonferroni adjusted *p*-value < 1 × 10^−10^ and absolute value of log_2_ fold change > 3.5) were selected (Supplementary Table 1). Unsupervised (agglomerative) hierarchical clustering using Spearman's correlation distance (1-correlation coefficient) as metric and average linkage clustering as linkage criterion showed that selected miRNAs were able to clearly discriminate GIST tissues from adjacent non-cancerous tissues (Figure 3).

### The composition of isomiRNAs in GIST differs from the adjacent gastric tissue

**Figure 4:**
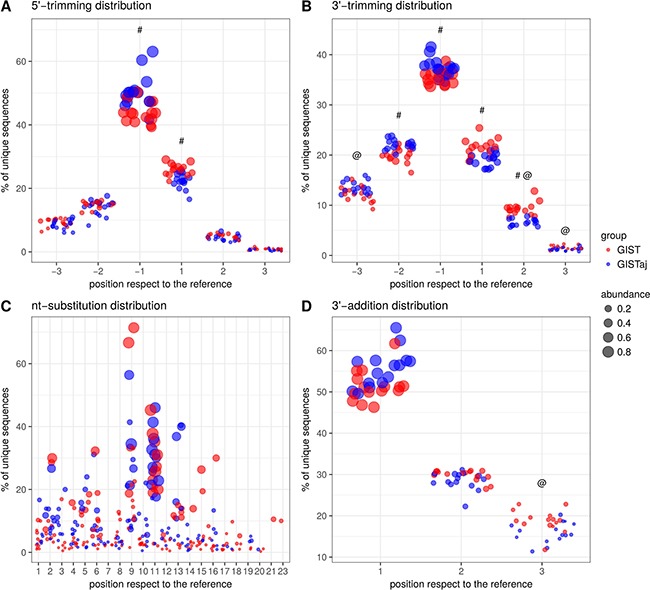
The distributions of isomiRNA modification types between GIST and adjacent tissue (**A**) 5′-trimming modification distribution. (**B**) 3′-trimming modification distribution. (**C**) nt-substitution modification. (**D**) 3′-addition modification distribution. @ - significant (Bonf. adjusted *p*-value < 0.05) differences of modification abundance values between GIST and adjacent tissue. # - significant differences of modification frequency between GIST and adjacent tissue.

To describe the variability of miRNAs in GIST, the analysis of isomiRNAs was performed on small RNA-seq data using mirAligner algorithm which enabled to identify variations at the 5′- and 3′-end and nucleotide (nt) substitutions. In order to increase reliability, only 2450 unique isomiRNAs having more than 10 read counts and more frequent than 1% in regards to the reference sequence have been selected for downstream analysis. Sequence variations were found in 87.98% of expressed miRNAs. The most common modification of isomiRNAs was 3′-trimming (75.43%, 1848/2450) followed by 3′-addition (44.33%, 1086/2450), 5′-trimming (32.00%, 784/2450), nt-substitution (4.12%, 101/2450). It is worth pointing out that 37.62% (38/101) of nt-substitutions occurred in the seed sites of miRNAs. More than one modification was observed in 44.33% (988/2450) of isomiRNAs. The overall differences in the distributions of isomiRNA modification types between GIST and adjacent tissue are shown in Figure 4 and Supplementary Table 2. To further identify the differences in the expression levels of miRNA variants, paired differential analysis was performed on isomiRNA count data. The analysis identified 219 deregulated isomiRNAs in 89 unique miRNA sequences (Bonferroni adjusted *p*-value < 0.01 and fold change > 3.5) between GIST and adjacent tissue (Supplementary Table 3). The majority of modifications in differentially expressed isoforms were in 3′-trimming (77.17%, 169/219) followed by 3′-addition (34.7%, 76/219) and 5′-trimming (25.57%, 56/219). There were no deregulated isoforms having nt substitutions neither in seed site nor in the whole sequence. The highest number of deregulated isoforms had miR-28-3p (*n* = 9), miR-483-3p (*n* = 9), miR-140-3p (*n* = 7), miR-192-5p (*n* = 7). Interestingly, 19 out of 21 miRNAs selected for validation study had deregulated isoforms.

### TLDA analysis verifies GIST miRNA expression profiles

In order to confirm the results of small RNA-seq, Taq-Man low-density array (TLDA) analysis was used to measure expression of the 21 selected mature miRNAs in the independent group of 40 paired GIST and adjacent non-cancerous tissue samples. Two-class paired differential expression analysis was applied on continuous expression data which was normalized to the mean levels of RNU6, RNU24 and RNU48. The expression levels of 19 miRNAs showed significant differential expression in the same direction as in the sequencing data (Bonferroni adjusted *p*-value < 0.01 and fold change > 2) and only two miRNAs (miR-483-3p and miR-378d) did not reach the significance threshold (Table 1, Supplementary Figure 1).

**Table 1:** Summary of miRNA validation results in GIST and adjacent non-cancerous tissues

miRNA ID	NGS data		TLDA data	
	log2 fold change	*p*-value (Bonferroni corrected)	log_2_fold change	*p*-value (Bonferroni corrected)
miR-133a-3p	−5.26	5.34 × 10^−25^	−6.33	4.12 × 10^−13^
miR-133b	−5.70	5.74 × 10^−26^	−8.92	4.45 × 10^−13^
miR-192-5p	−6.42	1.09 × 10^−25^	−7.63	6.33 × 10^−12^
miR-200c-3p	−7.38	4.25 × 10^−25^	−10.04	2.84 × 10^−11^
miR-200a-3p	−8.00	7.95 × 10^−24^	−10.97	3.28 × 10^−11^
miR-141-3p	−8.18	2.69 × 10^−30^	−10.50	4.69 × 10^−11^
miR-200b-3p	−8.27	4.75 × 10^−38^	−10.03	1.26 × 10^−10^
miR-486-5p	−4.92	1.86 × 10^−21^	−4.76	1.92 × 10^−10^
miR-429	−8.36	1.75 × 10^−25^	−9.38	3.70 × 10^−10^
miR-490-3p	−7.38	5.09 × 10^−22^	−5.37	5.39 × 10^−10^
miR-455-3p	3.81	5.15 × 10^−15^	5.67	7.56 × 10^−10^
miR-375	−9.07	4.77 × 10^−34^	−10.96	7.82 × 10^−10^
miR-203a-3p	−7.13	4.38 × 10^−25^	−8.49	8.37 × 10^−10^
miR-215-5p	−4.89	9.13 × 10^−15^	−5.81	1.02 × 10^−9^
miR-182-5p	−6.11	3.91 × 10^−34^	−5.00	2.77 × 10^−8^
miR-451a	−3.87	1.94 × 10^−14^	−4.69	4.12 × 10^−7^
miR-675-3p	5.87	2.49 × 10^−41^	2.88	9.78 × 10^−6^
miR-483-5p	5.04	2.31 × 10^−22^	3.26	1.41 × 10^−5^
miR-509-3p	8.42	1.36 × 10^−10^	4.70	0.0003
miR-483-3p*	6.28	1.94 × 10^−34^	1.43	0.15
miR-378d*	−3.62	1.12 × 10^−14^	−1.78	0.21

Differential expression was considered when adjusted *p*-value was < 0.01 and absolute value of log_2_ fold change was > 2. NGS data is presented for comparison. *Not validated.

### The mature miR-509-3p and miR-215-5p are associated with clinical characteristics of GIST

**Figure 5:**
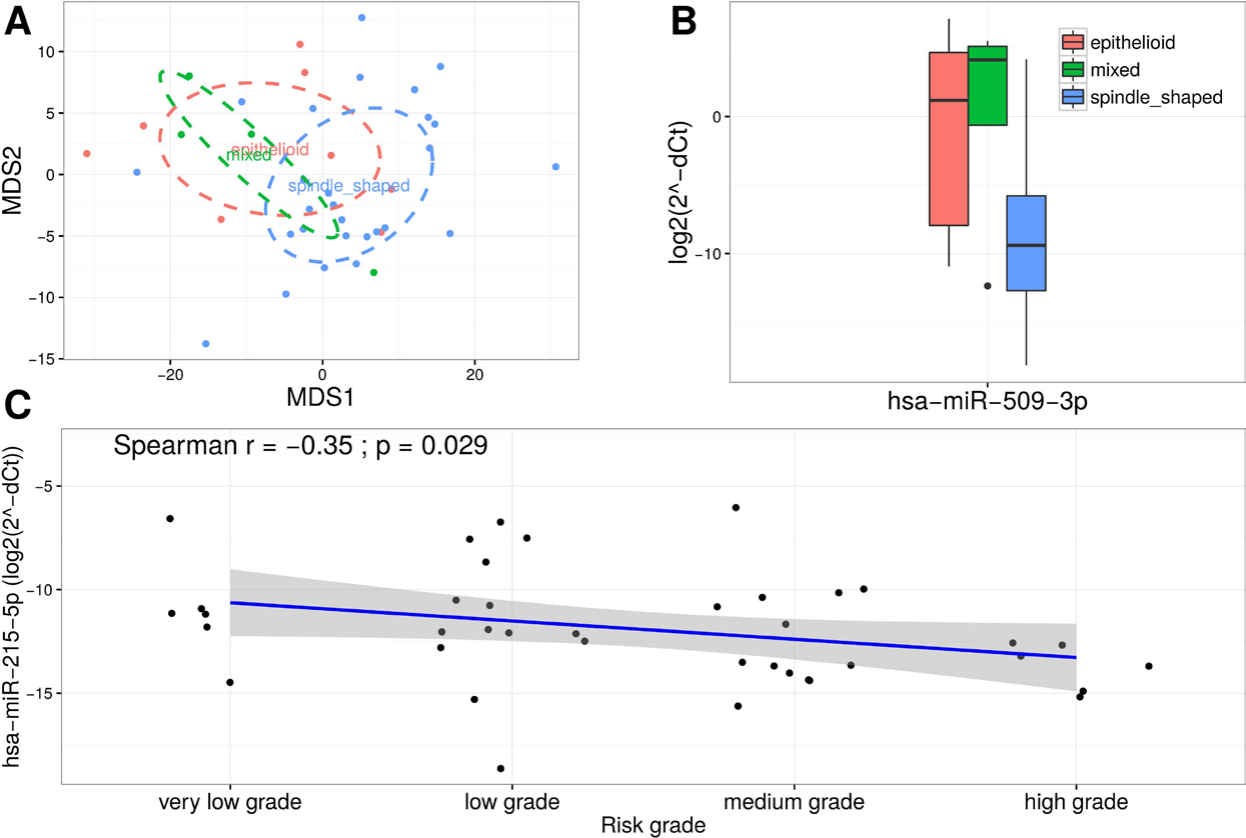
miR-509-3p and miR-215-5p are associated with clinical characteristics of GIST (**A**) MDS plot based on normalized TLDA data showing a slight clustering corresponding to GIST histological subtypes. (**B**) Box plots exhibiting the expression levels of miR-509-3p in different GIST histological subtypes. (**C**) Scatter plot showing moderate negative correlation between expression levels of miR-215-5p and risk grade of GIST.

To further investigate the clinical significance of validated miRNAs in GIST (replication group *n* = 40) subphenotype analysis based on histological *types* (spindle cell type (*n* = 27), epithelioid type (*n* = 8), and mixed spindle and epithelioid type (*n* = 4)) and disease risk grade (very low grade, low grade (*n* = 13), medium grade (*n* = 14) and high grade (*n* = 6)) was performed. MDS analysis based on the TLDA data revealed a slight clustering corresponding to GIST histological subtypes (Figure 5A). Therefore, multiple group comparisons were performed and significant up-regulation of miR-509-3p expression level (log_2_ fold change = 8.34; FDR adjusted *p*-value = 0.0001) in epithelioid and mixed types compared to spindle type (Figure 5B) were shown. To determine whether the expression levels of validated miRNAs were associated with the malignancy of GIST, Spearman's correlation analysis was performed. The analysis identified moderate negative correlation (*r* = −0.35, *p*-value = 0.029) between miR-215-5p and risk grade of GIST (Figure 5C). No further associations were found in the analysis.

### Enrichment analysis shows involvement of miRNAs in regulation of GIST related pathways

**Figure 6:**
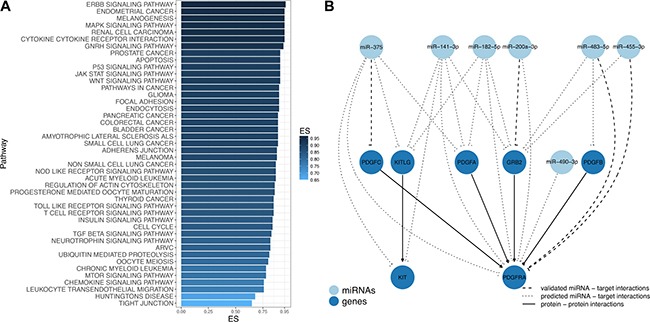
Differentially expressed miRNAs are involved in GIST-associated pathways (**A**) Overrepresented pathways in GIST identified by miRNA Set Enrichment Analysis. Bar chart showing the enrichment score (ES) of the significantly enriched (FDR *p*-value < 0.01) KEGG pathways. (**B**) Subnetwork of GIST-associated genes and validated miRNAs in GIST deregulated pathways. Each node represents miRNAs (light blue) and mRNA targets (blue), while edges exhibit different interactions between nodes: validated miRNA − mRNA target interactions (dashed line), predicted miRNA − mRNA target interactions (dotted line) and protein − protein interactions (solid line).

To further determine the possible biological impact of aberrantly expressed miRNAs in GIST pathogenesis, miRNA Set Enrichment Analysis on the set of miRNAs validated in TLDA analysis was performed. In total, 38 KEGG pathways were significantly enriched (FDR adjusted *p*-value < 0.01) with sets of miRNAs ranging from 10 to 16 molecules per enriched pathway (Figure 6A, Supplementary Table 4). Among the enriched biological pathways were cytokine-cytokine receptor interaction (hsa04060), ERBB signaling (hsa04012), p53 signaling (hsa04115), MAPK signaling (hsa04010), cell cycle (hsa04110), mTOR signaling (hsa04150), JAK/STAT signaling (hsa04630) and Insulin signaling (hsa04910) pathways, which were previously described as GIST-associated pathways [22]. In order to get deeper insights into the putative functionality of the enriched miRNAs, predicted and validated targets were retrieved and mapped to the above mentioned pathways. The results showed that well-known GIST-associated genes *KIT* and *PDGFRA* [1, 23] together or separately were involved in hsa04060 and hsa04010 pathways and were predicted targets for miRNAs comprised in the enriched sets (Figure 6B, Supplementary Table 5).

### Correlation analysis of miRNAs and their potential target genes in *KIT* and *PDGFRA*

Two GIST associated genes *KIT* and *PDGFRA* were predicted as potential targets for the enriched miRNAs. In order to support this data, first the expression analysis of target genes was performed in paired GIST and adjacent non-cancerous tissue samples. Both *KIT* and *PDGFRA* were found to be significantly upregulated in GIST tissue (log_2_ fold change 3.9 and 1.5, respectively, Bonferroni adjusted *p*-value < 0.01) (Supplementary Figure 2). Further, Pearson‘s correlation analysis was applied to unveil the relation between the *KIT* and *PDGFRA* and their targeting miRNAs expression levels in GIST cancerous and normal tissues. A high negative correlation was observed between *KIT* and miR-141-3p expression (*r* = −0.61, *p* = 2.88 × 10^−7^), while miR-375 exhibited a moderate negative correlation with KIT expression (*r* = −0.49, *p* = 8.75 × 10^−5^) (Supplementary Figure 3). Only one of the enriched miRNAs, miR-200a-3p, negatively correlated with *PDGFRA* gene expression with a designated significance level (*r* = −0.29, *p* = 0.022) (Supplementary Figure 3).

## DISCUSSION

Up to now, miRNA expression profiling in GIST has been performed using quantitative RT-PCR or microarray approaches, which allow to identify the differential expression of only limited number of known miRNAs [12, 18, 24]. This is the first study using small RNA-seq strategy for the discovery of GIST-relevant miRNAs in the phenotypically uniform study group, i.e. patients with tumor located in stomach.

Clearly different transcriptional miRNA profiles between GIST and adjacent non-cancerous tissues of the same patient have been demonstrated using small RNA-seq approach. Based on the statistical power of the study, strict selection criteria were applied and 110 miRNAs (34 of which were up-regulated and 76 were down-regulated) were identified to be differentially expressed in GIST. Thirty of them have been previously shown to be associated with GIST or different GIST phenotypes [12, 21, 25, 26]. Twenty one strongly deregulated and highly expressed miRNAs were selected for replication study and 19 miRNAs were verified to be associated with GIST. MiRNA selection criteria for the replication study were based on expression level of miRNAs, as more abundant miRNAs repress their mRNA targets to a greater degree [27–30]. Six of the replicated miRNAs, miR-133a-3p, miR-133b, miR-483-5p, miR-675-3p, miR-200c-3p, miR-509-3p, have been shown to be deregulated in previous GIST miRNA studies [17, 25, 31]. The remaining 13 miRNAs are novel GIST associations, but have been related with other oncogenic disorders, like colorectal cancer [32, 33], gastric cancer [34] and breast cancer [35]. Among the newly associated miRNAs are: miR-200 family members (miR-200c-3p, miR-200a-3p, miR-200b-3p, miR-429, miR-141-3) widely investigated as prognostic and diagnostic biomarkers for cancer [36]; miR-192/215 shown to be involved in cancer-related p53 network [37, 38]; and miR-375 shown to be down-regulated in gastric cancer and to affect cell proliferation [39]. Interestingly, two of the most extensively studied and previously validated miRNAs in GIST – miR-221 and miR-222 [26, 40] – were found to be significantly deregulated in our discovery cohort, but did not meet the selection criteria for validation study and were not further analyzed.

Moreover, small RNA-seq data revealed GIST related differences on composition and expression level of miRNA isoforms. The number of individual variants and their contribution to miRNA expression indicated that 3′-modified isomiRNAs were the predominant category. More than a third of the isomiRs had variations at the 5′-end or nt substitutions in positions 2–8, which result in a modified seed sequence. For these isomiRs, the contribution to the overall miRNA expression levels was lower, i.e. 5′- modification had a quarter of deregulated miRNA isoforms, whereas nt substitutions were not detected in any of the deregulated isoforms. It has been previously shown that seed-modifying variants have lower frequency and expression, however these isomiRNAs might alter mRNA target recognition and function [41, 42].

MiRNA enrichment analysis revealed that target genes of the validated miRNAs are involved in the main GIST associated signaling pathways like MAPK, ERBB, p53, mTOR, JAK/STAT, insulin pathway and cell cycle [43]. A subset of predicted or/and validated target genes of the here-investigated miRNAs, namely *KIT* [23] and *PDGFRA* [23], are known to be strongly involved in GIST biology. These miRNAs have been shown to be aberrantly expressed in many human malignant tumors and participate in various cellular processes. For example, miR-375, predicted to target *KIT* and *PDGFRA*, has been previously shown to inhibit cell growth through the AKT signaling pathway [44, 45]. These findings confirm putative involvement and importance of these miRNAs in GIST pathogenesis. Furthermore, our miRNA-mRNA correlation analysis revealed that three of the enriched downregulated miRNAs miR-375 and miR-141-3p were highly or moderately negatively correlated with an overexpressed target-gene *KIT* – a known GIST-associated oncogene. However, only one of the predicted enriched miRNAs miR-200a-3p showed a significant correlation to its target *PDGFRA*. These results support the miRNA-target-gene interactions predicted by bioinformatic tools.

Aberrantly expressed miRNAs were also tested for associations with clinical and histological characteristics. Expression level of miR-215-5p was found to be negatively correlated with the risk grade of the tumor and miR-509-3p was found to be up-regulated in epithelioid and mixed histological types compared to spindle type of GIST. Although miR-215-5p was not previously associated with development of GIST, it has been shown to be a tumor-suppressor and a biomarker of poor prognosis in colon and pancreatic cancers in several studies [46–48]. Another phenotypic association was detected for miR-509-3p which was significantly up-regulated in epithelioid and mixed histological types compared to spindle type of GIST in this study. Differential expression of miR-509-3p has previously been shown to be associated with mutation-dependent GIST [17]. No further associations were found in the analysis for other 17 validated miRNAs. However, sample size in the phenotypic subgroup analysis was small, therefore the results have to be replicated in a larger study group.

Our study has certain limitations that need to be acknowledged. First, the lack of ideal control group for GIST molecular studies, as this tumor originates from the interstitial cells of Cajal [49], which represent a very minor cell population in the normal intestinal tissue. Some previous studies have used leiomyosarcomas [5, 50], as a reference. Nevertheless, multiple molecular GIST studies have used adjacent tumor free tissue as controls [51–53]. Our study has also provided evidence that usage of adjacent histologically normal tissue as control groups reveals important deregulated microRNAs, which are predicted to regulate genes from GIST associated signaling pathways. Second, FFPE samples were used in this study, because of the limited availability of fresh-frozen GIST samples. However, previous studies have shown that miRNA profiles of FFPE samples correlate well with those obtained from matched frozen reference samples [54]. Third, we were not able to determine the prognostic value of miRNAs, as our study group had only a few individuals with metastatic tumors and relatively low GIST-dependent mortality rate. Nevertheless, we believe that our study adds valuable insights into the complex biology of GIST development.

In our study, using Illumina next-generation sequencing and TLDA technologies we provided strong evidence of 19 GIST-associated miRNAs, 13 of which were not previously reported as GIST associated miRNAs. Moreover, we showed that these newly identified miRNAs are involved in the main GIST associated pathways. miRNA-mRNA correlation analysis revealed a significant correlation between the all enriched miRNAs and their putative target gene *KIT*. Furthermore, phenotype analysis revealed miR-215-5p to be negatively correlated with the risk grade of GIST, while miR-509-3p were associated with epithelioid and mixed histological subtypes of GIST. Our findings provide a more detailed molecular understanding of GISTs, highly significant for further identification of new targets and development of novel therapeutics.

## MATERIALS AND METHODS

### Ethics and consent

The approval to perform the study was obtained from Kaunas Regional Biomedical Research Ethics Committee (No. BE-2–8). All patients have signed an informed consent form to participate in the study.

### Study population

55 patients diagnosed with GIST in the Hospital of Lithuanian University of Health Sciences during the years 2002–2015 were included in the study. All patients met the following selection criteria: histological confirmation of GIST diagnosis on surgical specimens; no previous specific GIST treatment. GIST diagnosis was based on tumor morphological features and positive c-kit/CD117 immunohistochemical reaction. In cases of negative CD117 immunostaining, diagnosis was made on the basis of morphological features, positive CD34 and negative desmin, alpha-smooth muscle actin (SMA), S100 immunostaining. Diagnosis was confirmed with genetic testing for *KIT, PDGFRA* and *BRAF* mutations (Supplementary Table 6). Three histological phenotypes were discriminated based on cell morphology: spindle, epithelioid (> 90% of a single cell type) and mixed types. Risk grade of tumors was assessed according to National Institutes of Health (NIH) GIST Consensus Criteria developed by Fletcher [55]. Characteristics of subjects are presented in a table below (Table 2). Distribution of all evaluated parameters was similar in both discovery and validation groups (*p*-value > 0.05). Fisher's exact test was used for qualitative measurements (i.e. gender, risk grade, etc.) and *t*-test was used for quantitative measurements (i.e. age).

**Table 2:** Summary of clinical and demographic characteristics of the GIST patients

	Discovery cohort (*N* = 15)	Validation cohort (*N* = 40)
**Age**		
Mean ± SD	62.80 ± 13.68	68.05 ± 10.32
**Gender, *N* (%)**		
Male	6 (40.0)	16 (40.0)
Female	9 (60.0)	24 (60.0)
**CD117, *N* (%)**		
Positive	15 (100.0)	37 (92.5)
Negative	0 (0.0)	2 (5.0)
Unknown	0 (0.0)	1 (2.5)
**Risk grade, *N* (%)**		
High	4 (26.7)	7 (17.5)
Moderate	4 (26.7)	13 (32.5)
Low	6 (40.0)	14 (35.0)
Very Low	1 (6.7)	6 (15.0)
**Metastasis, *N* (%)**		
Present	1 (6.7)	4 (10.0)
Absent	14 (93.3)	36 (90.0)
**Cell type**		
Spindle	12 (80.0)	28 (70.0)
Epithelioid	3 (20.0)	8 (20.0)
Mixed	0 (0.0)	4 (10.0)
**Mutational Status, *N* (%)**		
KIT exon 11	9 (60.0)	17 (42.5)
KIT exon 9	0 (0.0)	1 (2.5)
PDGFRA exon 18	2 (13.3)	9 (22.5)
PDGFRA exon 12	1 (6.7)	2 (5.0)
Wild type	3 (20.0)	3 (7.5)
Not detected	0 (0.0)	8 (20.0)

Evaluated parameters did not differ between compared groups, with estimated *p*-values > 0.05 (Fisher's exact and *t*-tests). SD – standard deviation.

### Molecular analysis of *KIT* and *PDGFRA*

*KIT* and *PDGFRA* mutation status in tumor samples was assessed by PCR using MyTaq HS DNA polymerase (Bioline, Germany) through 35 cycles with appropriate annealing temperatures. Primer sequences specific for *KIT* and *PDGFRA* mutations are shown in Supplementary Table 7. PCR samples were then subjected to direct sequencing of single-stranded PCR products using BigDye^®^ Terminator v1.1 cycle sequencing kit and the ABI Prism^®^ 310 genetic analyzer (Applied Biosystems). For *BRAF* mutation analysis the cobas^®^ 4800 BRAF V600 Mutation Test (Roche Diagnostics) was used according to the manufacturer's instructions.

### Tissue sample preparation and total RNA extraction

Total RNA was isolated from paired GIST tumor and adjacent histologically normal tissues, which were fixed in formalin and embedded in paraffin at a Department of Pathological Anatomy, Lithuanian University of Health Sciences. GIST tumor samples were dissected from non-necrotic, non-fibrotic zones with highest tumor cell count. Adjacent histologically normal tissue samples were dissected from tumor adjacent zones with no signs of histopathological changes. Paraffin was removed using xylene treatment followed by two steps of ethanol wash to remove remaining xylene. Further RNA extraction was performed using standard protocol of commercial miRNeasy FFPE Kit (Qiagen). Total RNA concentration was evaluated by Nanodrop2000 spectrophotometer (Thermo Scientific). Quality assessment of total RNA samples was performed using Agilent 2100 Bioanalyzer (Agilent Technologies).

### Preparation of small RNA libraries and next-generation sequencing

Small RNA libraries were prepared using TruSeq Small RNA Sample Preparation Kit (Illumina) according to the manufacturer's protocol with 1 μg RNA input per sample followed by RNA 3′ adapter ligation, RNA 5′ adapter ligation, cDNA synthesis, PCR amplification using unique barcode sequences for each sample and gel size-selection of small RNA library. The yield of sequencing libraries was assessed using the Agilent 2100 Bioanalyzer (Agilent Technologies). Multiplexed libraries were sequenced on HiSeq2500 (Illumina) next-generation sequencing platform.

### Bioinformatics analysis of small RNA-seq data

Several filtering steps were performed after obtaining and demultiplexing the raw reads. First, cutadapt [56] was used to trim low-quality ends of the reads with Phred quality score value (Q) > 30 and to remove 3′ adapter (5′-TGGAATTCTCGGGTGCCAAGG-3′) sequences from the reads. The trimmed sequences that were shorter than 17 bp were discarded. Second, in order to reduce computational time the reads with identical sequences were collapsed while saving the information about the read counts. Third, the collapsed reads which mapped to viral genome (RefSeq) [57] human tRNAs, rRNAs, snRNAs, sRNAs (Rfam) [58] and non-human miRNA precursors (miRBase v21) [59] were filtered using BLASTN v2.2.30 [60]. MiRNA and isomiRNA quantification in filtered reads were performed using the quantifier.pl module from miRDeep2 v2.0.0.7 software [61] and miraligner from SeqBuster software [62] with default parameters using precursor and mature miRNA sequences as reference (miRBase v21). Differential expression analyses of the size factor normalized counts of mature miRNAs and isomiRNAs between paired GIST and adjacent non-tumorous tissue samples were performed by use of negative binomial generalized linear models implemented in the R package DESeq2 [63]. The *p*-values resulting from *Wald tests* were corrected for multiple testing using Bonferroni correction. This study had 80% power to confidently identify (α = 0.01) differentially expressed miRNA genes with ~3.5 fold change (1.8 log2 fold change) or greater (Supplementary Figure 4). Therefore, miRNAs and isomiRNAs with a corrected *p*-value < 0.01, and fold change > 3.5 were considered to be significantly differentially expressed. Highly abundant (base mean > 100) and differentially expressed miRNAs with a corrected *p*-value < 1 × 10^−10^ and an absolute log2 fold change > 3.5 were selected for validation analysis. The statistical power of the study was calculated using the RNASeqPower package [64]. The small RNA-seq data have been deposited in NCBI's Gene Expression Omnibus [65] and are accessible through GEO Series accession number GSE89051 (https://www.ncbi.nlm.nih.gov/geo/query/acc.cgi?acc=GSE89051).

### Taq-Man low-density array (TLDA) analysis

Validation of miRNA expression profiles was performed using Custom TaqMan^®^ Array MicroRNA Cards (Applied Biosystems) which enabled to quantify 21 selected miRNAs and 3 reference genes (RNU6, RNU24 and RNU48) for data normalization. In short, 450 ng of total RNA was initially reverse transcribed using Megaplex RT pool. 1 μl of cDNA per port was loaded on 8-port Custom TaqMan^®^ Array MicroRNA Card and run on ViiA 7 Real-time PCR System (Applied Biosystems) following the standard protocol. All GIST samples were randomized and placed with their matching controls on the same TLDA card. The miRNA expression data in terms of continuous Ct values was generated by ViiA 7 Software (Applied Biosystems) with automatic settings for assigning the baseline. The miRNAs with a Ct value > 40 were considered unamplified. The samples which showed no variance were excluded from further analysis. TLDA expression data was normalized using the ΔΔCT method [66] to mean expression values of RNU6, RNU24 and RNU48. Batch effects, identified using Kruskal-Wallis tests, were corrected using the ComBat function from the sva package [67] (Supplementary Figure 5). *C*orrected miRNA expression data of GIST and adjacent non-tumorous tissue samples were compared using paired Mann-Whitney U test (Wilcoxon rank-sum test). MiRNAs with a Bonferroni-corrected *p*-value < 0.01 and a fold change > 2 were considered to be significantly differentially expressed. Data analysis was performed using the HTqPCR package [68].

### Enrichment analysis

Enrichment analysis was performed using miRNA set enrichment analysis method which is based on widely used GSEA [69] and implemented in the MiRSEA package [70]. The gene sets of pathways were obtained from the KEGG database and the gene sets of miRNA targets were obtained from TarBaseV6.0, mir2Disease, miRecords and miRTarBase. The pathways with FDR corrected *p*-value < 0.01 were considered to be significantly deregulated. For the enriched set of miRNAs, predicted and validated targets were retrieved using the multiMiR [71] package and visualized in deregulated pathways using the KEGGgraph [72] and Rgraphviz [73] packages.

### Phenotypic association analysis

MiRNA expression and GIST phenotype association analysis was performed by multiple group comparisons using Limma *t*-test from the HTqPCR package [68] and Spearman correlation analysis using the Hmisc package [74]. All statistical analyses were performed using the statistical computing environment R (version 3.2.3) [75].

### Correlation analysis of miRNAs and their potential target genes in *KIT* and *PDGFRA*

Expression levels of predicted target genes were detected by quantitative PCR. Total RNA, extracted from FFPE tissue (method described above), was reverse transcribed using TaqMan High Capacity cDNA Reverse Transcription kit (Applied Biosystems). The expression of human *KIT, PDGFRA*, *TFRC* and *POLR2A* genes was detected using TaqMan Gene Expression Assays and the TaqMan Universal Master Mix according to the manufacturers recommendations using 7500 Fast Real-Time PCR system (Applied Biosystems). The expression data was normalized to *TFRC* and *POLR2A* reference genes, which were previously described as the most suitable reference genes for GIST [76]. Spearman correlation was applied on ΔCt data. *P*-value below 0.05 was considered statistically significant.

## SUPPLEMENTARY MATERIALS FIGURES AND TABLES














