# Supplementary material for: MiRNA profiling of gastrointestinal stromal tumors by next-generation sequencing
Source: Oncotarget. 2017 Mar 29;8(23):37225–38. doi: 10.18632/oncotarget.16664 (PMC5514905; doi:10.18632/oncotarget.16664)
Supplement: Supplementary file 1 [file oncotarget-08-37225-s001.pdf]

MiRNA profiling of gastrointestinal stromal tumors by next generation sequencing

Supplementary Materials

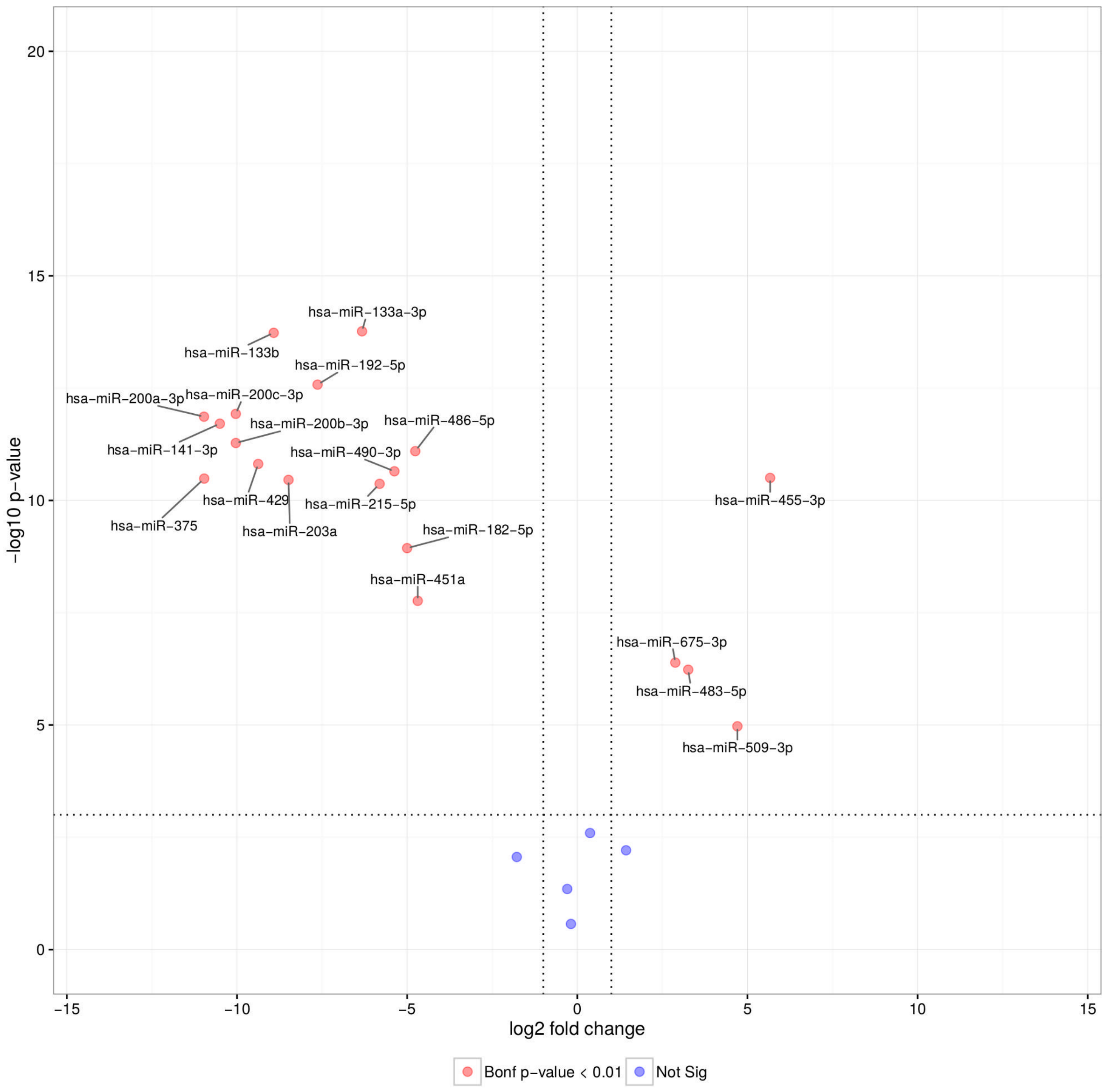

Supplementary Figure 1: Differential expression of validated miRNAs in GIST detected by TLDA. The red color represents significantly (Bonferroni adjusted $p < 0.01$) differentially expressed miRNAs with fold change < 2, while blue color represents non-differentially expressed miRNAs.

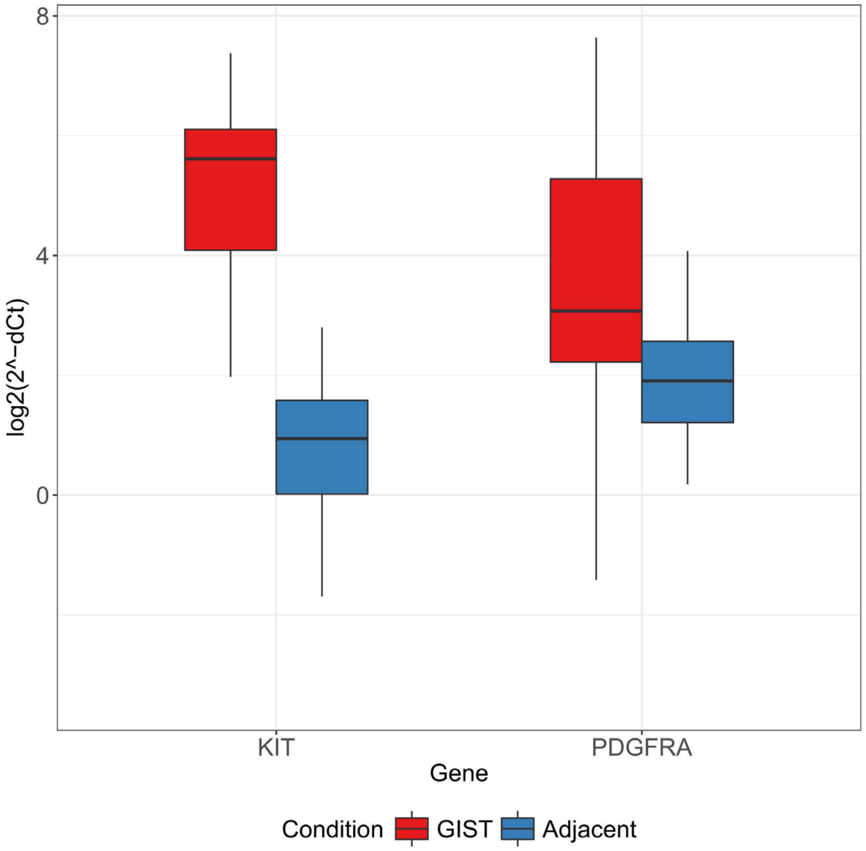

Supplementary Figure 2: Differential expression of KIT and PDGFRA in GIST and adjacent tissues. Expression levels of *KIT* and *PDGFRA* genes were analyzed using qRT-PCR. The data is represented as $\log_2 2^{(-\Delta Ct)}$.

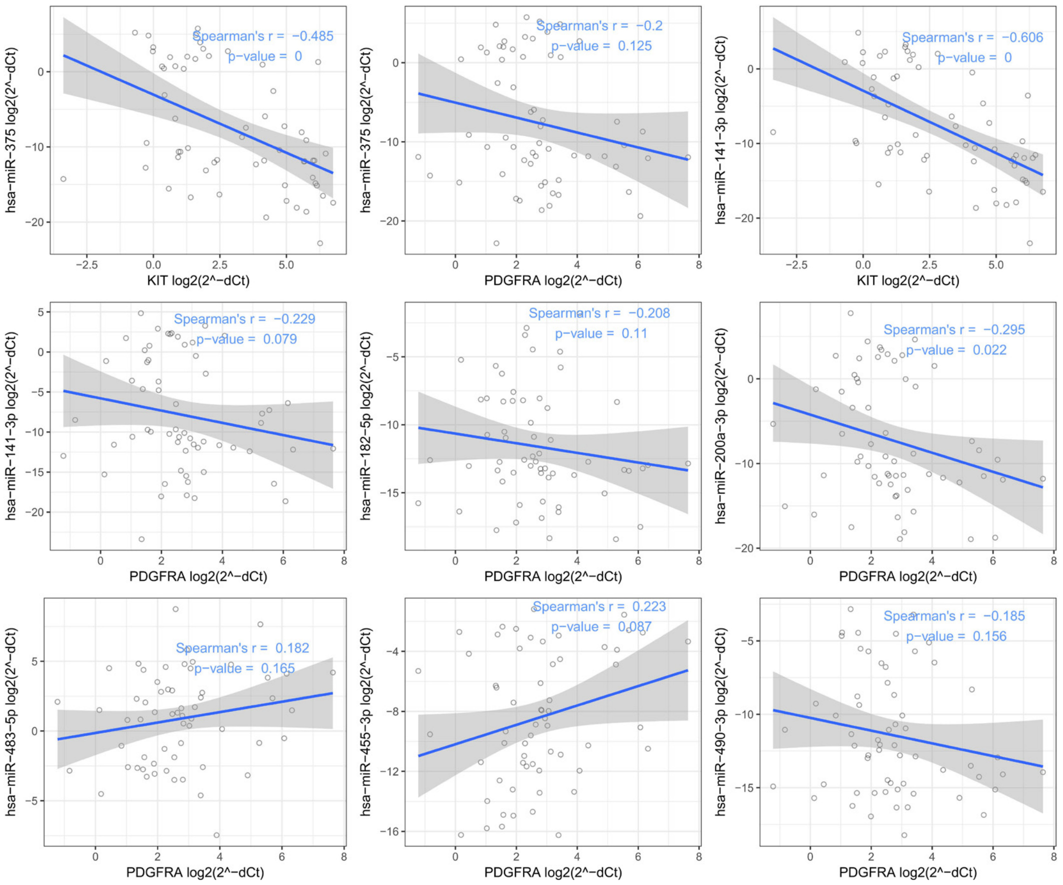

Supplementary Figure 3: Correlation analysis of miRNAs and their potential target genes *KIT* and *PDGFRA*. Spearman correlation coeafficient was calculated using normalized microRNA and mRNA expression data. *P*-value below 0.05 was considered significant.

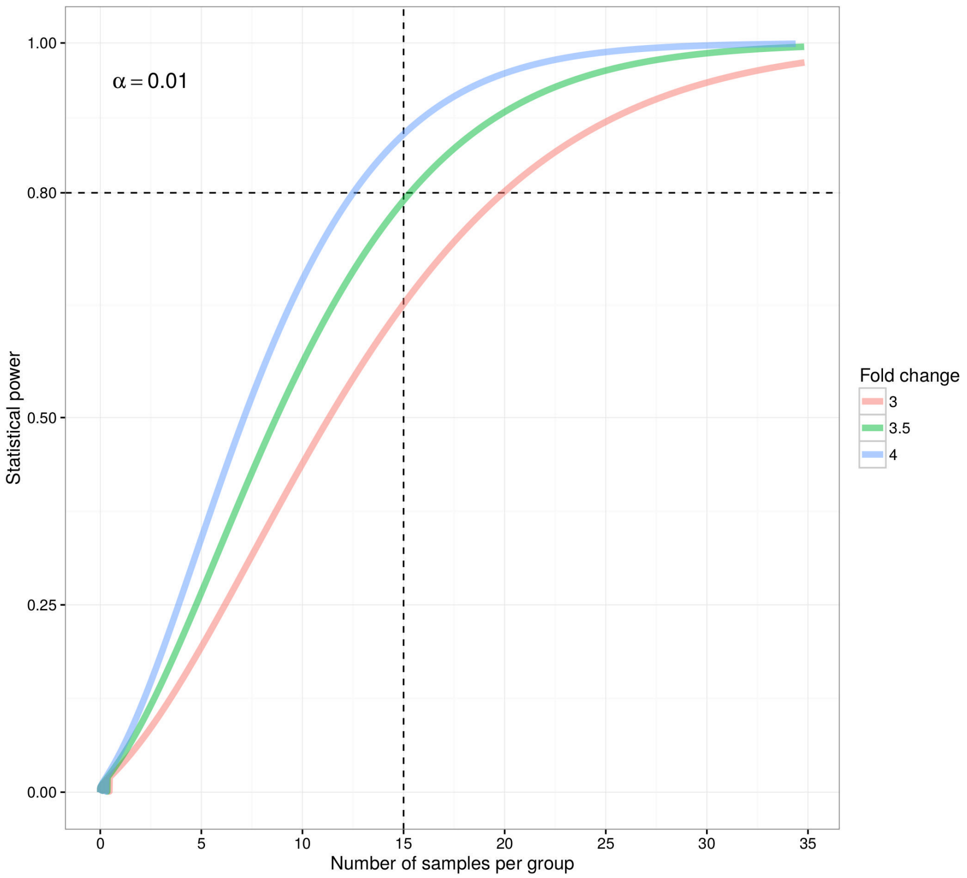

Supplementary Figure 4: Statistical power of the study. The colored lines show statistical powers for different effect size (fold change). The intersection of vertical and horizontal lines show the significantly ($p < 0.01$) detectable effect size with 80% statistical power in this study.

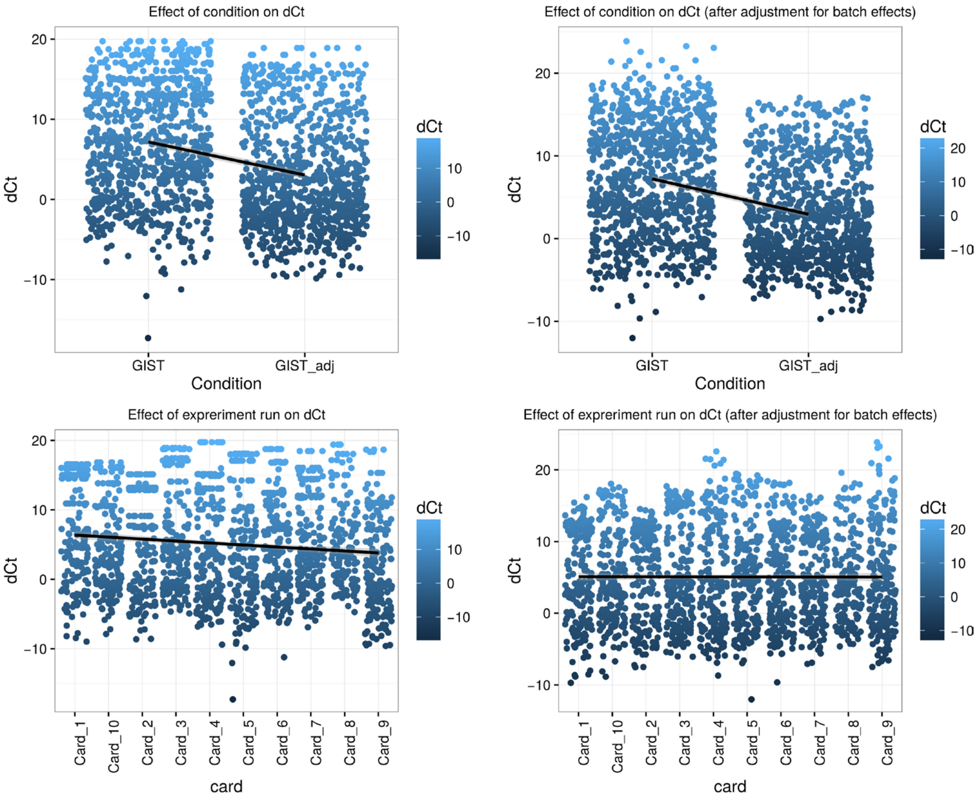

Supplementary Figure 5: TLDA data correction for batch effects using combat. Plots on the left side show ΔCT values before the correction, while plots on the right side show ΔCT values after the correction.

Supplementary Table 1: Differentialy expressed miRNAs between GIST and adjacent non-cancerous tissues (NGS data). See Supplementary_Table_1

Supplementary Table 2: Differences in the distributions of isomiRNA modification types between GIST and adjacent tissue. See Supplementary_Table_2

Supplementary Table 3: Differentialy expressed isomiRNAs between GIST and adjacent non-cancerous tissues (NGS data). See Supplementary_Table_3

Supplementary Table 4: Results of microRNA Set Enrichment Analysis. See Supplementary_Table_4

Supplementary Table 5: Validated miRNAs targeting GIST-associated genes KIT and PDGFRA. See Supplementary_Table_5

Supplementary Table 6: KIT, PDGFRA and BRAF mutational status in GIST. See Supplementary_Table_6

Supplementary Table 7: Primer sequences specific for KIT exons 9, 11, 13 and PDGFRA exons 12, 18

Primer	Forward	Reverse
KIT ex9	5′-CTAGAGTAAGCCAGGGCTTTTGTT-3′	5′-CCTAAACATCCCCTTAAATTGGATT-3′
KIT ex11	5′-GTGCTCTAATGACTGAGAC-3′	5′-TACCCAAAAAGGTGACATGG-3′
KIT ex13	5′-TTTTGCTAAAATGCATGTTTCCA-3′	5′-TAAAAGGCAGCTTGGACACG-3′
PDGFRA ex12	5′-TGGTGCACTGGGACTTTGGTA-3′	5′-AAAGGGAGTCTTGGGAGGTTACC-3′
PDGFRA ex18	5′-CAGGGGTGATGCTATATCAGCTACA-3′	5′-GTCCAGTGTGGGAAGTGTGGAC-3′
